# Apologetic

**Published:** 1857-03

**Authors:** 


### APOLOGETIC.

*Gentlemen of the Profession:*

All of those articles on Dental Practice that you were to send in for publication in the Register, have not as yet appeared. Well, gentlemen, really you must excuse us for the delay, for the fact is, they have not come to hand. Well, it has been a hard winter, and things generally froze up, and probably, ideas have been in the same fix; but it has thawed out now, and we should not be astonished if it rained articles for the Register. Come, tell us how you fill teeth. Do you meet with any difficulties? if so, what are they? How do you keep your cavities dry in all cases?

Do you ever have any failures? Some operators never *make* any failures. Well, they come fast enough without *making.*

